# miR-143 promotes angiogenesis and osteoblast differentiation by targeting HDAC7

**DOI:** 10.1038/s41419-020-2377-4

**Published:** 2020-03-09

**Authors:** Renkai Wang, Hao Zhang, Wenbin Ding, Zhenyu Fan, Boyao Ji, Chen Ding, Fang Ji, Hao Tang

**Affiliations:** 1 Department of Orthopedics, Changhai Hospital, Second Military Medical University, Shanghai, China; 2 Guangdong Key Lab of Orthopedic Technology and Implant Materials, Key Laboratory of Trauma and Tissue Repair of Tropical Area of PLA, Hospital of Orthopedics, General Hospital of Southern Theater Command of PLA, 111 Liuhua Road, Guangzhou, Guangdong 510010 China

**Keywords:** Cell lineage, Osteoporosis

## Abstract

The regulation of bone formation and detailed mechanisms are still largely elusive, and the roles of microRNAs in this process have attracted much attention. Recently, a specific subtype of CD31^hi^endomucin^hi^ (CD31^hi^EMCN^hi^) endothelium has been identified to promote bone formation, together with osteoblast development. However, the role of microRNA143 in the generation of CD31^hi^ EMCN^hi^ endothelium and bone formation remains unknown. In this study, we found that miR-143 was expressed both in osteoblast cells and CD31^hi^EMCN^hi^ endothelial cells. Serum miR-143 level was negatively correlated with age in humans. Overexpression of miR-143 promoted osteoblast formation and angiogenic effects. Furthermore, CD31^hi^Emcn^hi^ vessels and osteoblast formation were significantly inhibited in miR-143 knockout mice. Mechanistically, inhibitor HDAC7 was directly targeted by miR-143 and knockdown of HDAC7 was found to rescue the function of miR-143 deficiency. Thus, miR-143 promotes angiogenesis coupling with osteoblast differentiation by targeting HDAC7, which may serve as a potential target in angiogenic and osteogenic diseases.

## Introduction

Bone formation is directly regulated by the number and function of osteoblasts and osteoclasts^1–3^. Osteogenesis is under the regulation of several signaling pathways, including the transforming growth factor-β pathway and the receptor activator of the nuclear factor-κB (NF-κB) ligand (RANKL; also known as TNFSF11), the receptor activator of the NF-κB (RANK) pathway^4,5^, which involve several transcription factors, such as Runt-related transcription factor 2 (Runx2), nuclear factor of activated T cells, and Osterix (Osx)^6,7^. Runx2 is the vital determinant of osteoblast differentiation and its inactivation delays osteoblast differentiation, whereas the low-density lipoprotein receptor-related protein 5 signaling pathway controls osteoblast proliferation. Therefore, Runx2 is the key to osteoblastic bone formation and is accurately regulated by a set of mechanisms, such as Osx, which acts downstream of Runx2^3^. However, the regulation of Runx2 activation on osteoblast differentiation remains not fully determined.

Recently, a specific vessel subtype of endothelium—H-type vessels (CD31^hi^EMCN^hi^)—was identified to provide specific molecular signals that promoted bone formation^8^. CD31^hi^EMCN^hi^ endothelium formed an appropriate niche for both osteoblast and osteoclast development. These H-type vessels can also secrete Noggin to mediate osteoprogenitors differentiation^9^. When in a hypoxic state, the number of H-type vessels can be increased by the stimulation of Hypoxia Inducible Factor 1 (HIF-1) through NOTCH-DLL4 pathway signals^9^. However, the other signaling pathways that alter the number of CD31^hi^EMCN^hi^ endothelium are still not fully demonstrated.

MicroRNAs (miRNAs) are single-stranded noncoding ~22-nucleotide small RNAs that are involved in the regulation of gene processing and biological processes, and regulate mRNA expression by binding 3′-untranslated regions (3′-UTRs)^10–13^. A series of miRNAs have been identified to participate in osteogenic differentiation and osteoblastic bone formation. The deletion of Dicer alleles in mice at different stages of bone formation has confirmed that miRNAs control every step of osteogenesis. However, the roles of miRNAs in the regulation of bone formation, including osteoblast differentiation and type-H vessels, are still unclear.

Histone deacetylases (HDACs) are a family of enzymes that are involved in gene transcriptional regulation by removing acetyl groups from lysine residues on histones^14–16^. Several HDACs have been reported to contribute to bone development. For example, class II HDACs repress Runx2 function through Smad3^17^, HDAC7 and Runx2 have been found to be colocalized in nuclei, and HDAC7 is associated with Runx2 in osseous cells. Furthermore, HDAC7 represses Runx2 transcriptional activity in a deacetylase-independent manner and suppresses osteoblast maturation^19^. HDAC7 can also control endothelial cell growth through β-catenin. However, the interaction between HDACs and bone formation, including the regulation of CD31^hi^Emcn^hi^ endothelial cells, still needs investigation.

In this study, we found that miR-143 is an important miRNA in osteoblast differentiation and in CD31^hi^Emcn^hi^ endothelial cells through miRNA sequencing analysis. Furthermore, we found that the expression levels of miR-143 in serum from aged patients with fractures were lower than those of younger patients. Therefore, we generated miR-143-knockout mice using CRISPR/Cas9 technology and found that bone formation was inhibited in miR-143-knockout mice. The administration of agomiR-143 in vivo promoted osteoblastic bone formation and prevented bone loss in age-related osteoporosis. Therefore, we focused on the roles of miR-143 in bone formation in this study.

## Materials and methods

### Preparation of human serum

We collected human serum from 42 patients with fractures aged between 30 and 100 years from Shanghai Changhai Hospital, Second Military Medical University. Patients who had fractures caused by falling without obvious signs of violence were included in our study (inclusion criteria). Subjects with cancer, diabetes, or other severe diseases within the previous 5 years were excluded from our study (exclusion criteria). The clinical study was approved by the Committees of Clinical Ethics of the Shanghai Changhai Hospital, Second Military Medical University, and we obtained informed consent from the participants.

### Mice

C57BL/6 mice (4–6 weeks old) were obtained from Joint Ventures Sipper BK Experimental Animal Co. miR-143-knockout mice were generated using CRISPR/Cas9 technology, which harbors a 716 bp deletion flanking miR-143 loci, and was constructed by a service provider (ViewSolid Biotech) with the details described in Fig. 3a. The primers for mouse identification were forward: 5′-GCA GGG TGA GTA GGT GGT TTG-3′ and reverse: 5′-GTG TGG TTC TGT CTC TGC TGT TAC T-3′. All animal experiments were undertaken in accordance with the National Institute of Health Guide for the Care and Use of Laboratory Animals, with the approval of the Scientific Investigation Board of Second Military Medical University (Shanghai, China). The mice were housed in barrier housing conditions at the animal center of Second Military Medical University. Mice were injected with calcein (10 mg/ml, 30 mg/kg/mouse) twice at 10 days and 3 days before bone collection.

### Reagents

AgomiR-143, the respective negative control (AgomiR-NC), HDAC7-siRNA for in vitro RNA interference, cholesterol-conjugated HDAC7-siRNA for in vivo RNA interference, and their respective negative controls were obtained from RiboBio, Guangzhou, China.

### Differential miRNA expression analysis

DEGseq v1.18.0 was used for differential gene expression analysis between two samples with nonbiological replicates. Under the assumption that the number of reads derived from an miRNA follows a binomial distribution, DEGseq was used based on MA-plot and widely used for differential gene expression analysis. A *p*-value was assigned to each gene and adjusted by Benjamini and Hochberg’s approach for controlling the false discovery rate. miRNA with *p* < 0.05 and log_2_ fold change > 1 were identified as differentially expressed miRNAs (DEMs). DESeq (v1.16.0) was used for differential gene expression analysis between two samples with biological replicates using a model based on a negative binomial distribution. A *p*-value was assigned to each miRNA and adjusted by Benjamini and Hochberg’s approach for controlling the false discovery rate. miRNA with *p* < 0.05 and log_2_-fold change > 1 were identified as DEMs.

### Differential gene expression analysis

DEGseq was used for differential gene expression analysis between two samples with nonbiological replicates. Under the assumption that the number of reads derived from a gene (or transcript isoform) follows a binomial distribution, DEGseq was used based on MA-plot and widely used for differential gene expression analysis. A *p*-value was assigned to each gene and adjusted by Benjamini and Hochberg’s approach for controlling the false discovery rate. Genes with *p* < 0.05 and log_2_ fold change > 1 were identified as DEGs. DESeq2 was used for differential gene expression analysis between two samples with biological replicates under a theoretical basis and obeys the hypothesis of a negative binomial distribution for the value of counts. In contrast to DESeq, DESeq2 estimates the depth parameters of samples and gene-specific parameters and uses linear regression for dispersion to minimize values, which was mainly considered for genes with the same expression level, which may share similar deviations or have their expression characteristics. DESeq2 estimates the expression level of each gene per sample by linear regression and then calculates the *p*-value with the Wald’s test. Finally, the *p*-value was corrected by the Benjamini-Hochberg (BH) method. Genes with *p* < 0.05 and log_2_-fold change > 1 were identified as DEGs.

### Flow cytometry

Femur and tibiae were dissected from miR-143-knockout mice and wild-type (WT) mice. Then we crushed the metaphysis and diaphysis regions of the bone in phosphate-buffered saline (PBS) (Gibco, pH 7.4) for the bone marrow and digested it with 2.5 mg/ml Collagenase (Sigma) at 37 °C for 30 min to obtain single-cell suspensions. After washing, the cells were blocked with Allophycocyanin (APC)-conjugated EMCN antibody (eBioscience), PE/Cy7-conjugated CD31 (Biolegend), fuorescein isothiocyanate (FITC)-conjugated CD45 (Biolegend), and FITC-conjugated Ter119 (Biolegend) at 4 °C for 30 min. After washing, the cells were performed on a Fortesa FACS cytometer system (BD Bioscience) and analyzed using FlowJo software.

### μCT analysis

Femur samples dissected from mice were scanned and analyzed by micro-CT (Quantum GX, PE). The scanner was set at a voltage of 80 kV, a current of 500 μA and a resolution of 16 μm per pixel. Image reconstruction software (NRecon v1.6), data analysis software (CTAn v1.9), and three-dimensional model visualization software (μCTVol v2.0) were used to analyze the parameters of the distal femoral metaphyseal trabecular bone. The trabecular bone region of interest was selected from 5% of the femoral length proximal to the distal epiphyseal growth plate and extended proximally to 0.5 mm, to measure the trabecular bone volume per tissue volume, trabecular number, trabecular separation, and trabecular thickness.

### Immunofluorescence staining

To examine dynamic bone formation, we subcutaneously injected 0.1% calcein (Sigma-Aldrich, 10 mg/kg body weight) in PBS into mice 10 and 3 days before killing the mice. We observed calcein double labeling in undecalcified bone slices under a fluorescence microscope. We measured trabecular bone formation in four random fields in the distal metaphysis of the femur.

Fresh bone tissues dissected from miR-143-knockout mice and WT mice were fixed in 4% paraformaldehyde solution overnight and decalcified with 0.5 M EDTA with constant shaking. All the bone tissues were embedded in OCT (Sakura). Thirty-micrometer bone sections were stained with antibodies CD31 (Santa Cruz, 1:50) and endomucin (Santa Cruz, 1:50). Then, we used secondary antibodies conjugated with fluorescence (Santa Cruz, 1:500). Nuclei were counterstained with 4′,6-diamidino-2-phenylindole. we observed the sections under a confocal microscope.

### Cell culture

We collected bone marrow from tibiae and femurs from 4-week-old male WT or miR-143-knockout mice and then digested it with collagenase (Sigma, 2.5 mg/ml) to obtain a single-cell suspension. Endothelial cells were sorted using endomucin antibody (Santa Cruz, 1:50) and then cultured in endothelial cell growth medium (Gibco). The cells were maintained at 37 °C in a 5% CO_2_ humidified incubator.

MC3T3-E1 cells were cultured in α-MEM (Gibco, USA) supplemented with 10% fetal bovine serum (FBS) (Gibco, USA) and 1% penicillin–streptomycin (Gibco, USA). We collected bone marrow mesenchymal stem cells (BMSCs) from 4-week-old male WT or miR-143-knockout mice and BMSCs were cultured in α-MEM (Gibco, USA) supplemented with 10% FBS (Gibco, USA) and 1% penicillin–streptomycin (Gibco, USA). After 48 h, we removed nonadherent cells and cultured the adherent cells for an additional week with a single media change. The cells were maintained at 37 °C in a 5% CO_2_ humidified incubator.

### In vitro transfection

BMSC cells or bone marrow endothelial cells (BMECs) or MC3T3-E1 cells (2 × 10^5^) were seeded into each well of six-well plates and incubated overnight, and then transfected with RNAs using Lipofectamine RNAiMAX (Thermo Fisher Scientific) as described previously^11^.

### In vivo transfection

All animal experiments were conducted in accordance with the National Institutes of Health Guide for the Care and Use of Laboratory Animals, with the approval of the Scientific Investigation Board of the Second Military Medical University (Shanghai, China). For delivery of cholesterol-conjugated HDAC7-siRNA, 10 nmol RNA in 0.1 ml saline buffer was locally injected into bone marrow once every 3 days for 2 weeks. For therapeutic overexpression of miR-143 in aged mice, 12-month-old female mice were treated with agomir-143 (10 mg/kg body weight) or the negative control agomir (Agomir-NC) by tail vein injection once every 3 days for 2 weeks.

### In vitro osteoblast differentiation

To induce osteoblast mineralization, MC3T3-E1 cells or BMSCs were seeded in six-well plates and a commercial kit was used (Cyagen) to differentiate the cells into osteoblast. The medium was supplemented with 100 µM miR-143 mimics or 100 µM miR-143 inhibitors (Guangzhou RiboBio Co., Ltd) and changed every 2 days. At day 21, the cells were treated with Alizarin red stain to analyze mineralization activity.

### Alizarin red staining

Cells were fixed in 4% ice-cold paraformaldehyde for 30 min and rinsed with ddH2O (double distilled H2O). Cells were stained with 40 mM Alizarin red S (Sigma-Aldrich) at pH 4.0 for 10 min with gentle agitation. The cells were washed five times with ddH2O and then washed five to six times with 1× PBS with gentle agitation.

### Alkaline phosphatase staining

Cells were fixed in 4% ice-cold paraformaldehyde for 30 min and rinsed with ddH2O (double distilled H2O), and stained for alkaline phosphatase (ALP) expression using BCIP/NBT staining kit (Beyotime, China). The cells were washed five times with ddH2O and then washed three to four times with 1× PBS with gentle agitation.

### RT-PCR and real-time PCR

Total RNA from bone tissues or cultured cells was isolated using TRIzol reagent (Life Technologies, USA) and complementary DNAs (cDNAs) were synthesized from 1 μg of total RNA using a high-capacity cDNA reverse transcription kit (Applied Biosystems, USA). Then, quantitative reverse transcriptase PCR (qRT-PCR) was performed using FastStart Universal SYBR Premix ExTaq^TM^ II (Takara Biotechnology, Japan) on an ABI Prism 7900 HT Sequence detection system (Applied Biosystems, USA). The primers for mRNAs were purchased from Sangon Biotech Co., Ltd, Shanghai. The primer sequences used for qRT-PCR were as follows: Alp: forward, 5′-CCA ACT CTT TTG TGC CAG AGA-3′ and reverse, 5′-GGC TAC ATT GGT GTT GAG CTT TT-3′; BGLAP: forward, 5′-CTG ACC TCA CAG ATG CCA AGC-3′ and reverse, 5′-TGG TCT GAT AGC TCG TCA CAA G-3′; HDAC7: forward, 5′-TGA AGA ATG GCT TTG CTG TG-3′ and reverse, 5′-CAC TGG GGT CCT GGT AGA AA-3′; mmu-miR-143–3p: forward, 5′-GGG GTG AGA TGA AGC ACT G-3′ and reverse, 5′-CAG TGC GTG TCG TGG AGT-3′; GADPH: forward, 5′-CAC CAT GGA GAA GGC CGG GG-3′ and reverse, 5′-GAC GGA CAC ATT GGG GGT AG-3′.

### Tube formation assay

One hundred microliters of Matrigel was transferred into a 48-well plate (per well) at 37 °C for 1 h and then BMECs were seeded into each well of this 48-well plate and transfected with RNAs using Lipofectamine RNAiMAX (Thermo Fisher Scientific) for 48 h^18^. Tube formation was observed under an inverted microscope. The total branching points, total tube length, and total loops were measured by using Image-Pro Plus 5.0 software.

### Western blot analysis

Cells were lysed using M-PER Protein Extraction Reagent (Thermo Fisher Scientific) supplemented with a protease inhibitor cocktail (Thermo Fisher Scientific). Protein concentrations were measured with a BCA assay (Thermo Fisher Scientific) and normalized to the extraction reagent. Equal amounts of the extracts were loaded and subjected to SDS-polyacrylamide gel electrophoresis (PAGE), transferred onto nitrocellulose membranes, and then blotted as reported. Antibodies specific to mouse GADPH (CST, 5174), HDAC7 (CST, 10831), RUNX2 (CST, 12556), and secondary antibodies were purchased from Cell Signaling Technologies.

### mRNA 3′-UTR cloning and luciferase reporter assay

The HDAC7 3′-UTR luciferase reporter construct was constructed by amplifying the mouse HDAC7 mRNA 3′-UTR sequence (including the predicted miR-143 binding site) by PCR. We purified the PCR products and cloned it into the XbaI site of the pGL3-promoter vector (Promega) immediately downstream of the stop codon of luciferase. We prepared the Hdac7 mutations by using a QuikChange Site-Directed Mutagenesis Kit (Stratagene) to get MUT-pGL3-Hdac7, which is confirmed by sequencing. MC3T3-E1 cells and BMECs were transfected with either WT or mutant pGL3 construct and 40 ng of pRL-TK-*Renilla*-luciferase plasmid and miR-143 mimics or inhibitors for 48 h. Luciferase activities were measured using the Dual-Luciferase Reporter Assay System (Promega) according to the manufacturer’s instructions. Luminescent signals were quantified by a luminometer (Promega), and each value from the firefly luciferase construct was normalized to Renilla luciferase activity.

### Scratch wound assay

BMECs were seeded into each well of this 48-well plate and transfected with RNAs using Lipofectamine RNAiMAX (Thermo Fisher Scientific) for 48 h and cultured until conference^18^. Next, we wounded the confluent cells by scratching the monolayer under an inverted microscope. Images of the cells were obtained straightway, 3 h and 12 h later. The rate of migration area and migration area (%) were calculated as described previously^18^.

### Transwell migration assay

BMECs were seeded into each well of 6-well plate and transfected with RNAs using Lipofectamine RNAiMAX (Thermo Fisher Scientific) for 48 h. Then, BMECs (2 × 10^4^) were seeded into the top chamber of a 24-well, 8 μm pore-size transwell plate (Corning). Then, complete medium was added to the bottom chamber. Twelve hours later, we removed the unmigrated cells by wiping the membranes with cotton swabs and stained the migrated cells with 0.5% crystal violet for 3 min and counted under an optical microscope.

### Northern blottings

Small RNAs (~200 nt) were enriched from Trizol (Thermo Fisher Scientific)-extracted total RNA using miRNeasy Mini kit (QIAGEN), according to the manufacturer’s instruction. Forty micrograms of small RNAs was dissolved in 2 × RNA loading buffer (Takara), heated at 95 °C for 5 min, loaded onto 7 M urea-16% PAGE gel, transferred to Zeta-Probe GT membrane (Bio-Rad), and cross-linked with ultraviolet irradiation (1200 mJ/cm^3^). The membranes were subjected to hybridization with biotin-labeled DNA oligonucleotide probes for miR-143 and U6 small nucleolar RNA (snRNA) overnight at 42 °C, and the detection of hybridization signal was performed using Chemiluminescent Nucleic Acid Detection Module Kit (Thermo Fisher Scientific) and imaged using ECL system (Bio-rad). The biotin-labeled DNA oligonucleotide probe sequences for miR-143 and U6 snRNA (loading control) were 5′-TGA GCT ACA GTG CTT CAT CTC A-3′ and 5′-TGT GCT GCC GAA GCG AGC AC-3′.

### Statistical analysis

All data are reported as the mean ± SD. Two-tailed Student’s *t*-test was used for comparisons between two groups and one-way analysis of variance was used for comparisons between multiple groups. For all experiments, *p < 0.05 was considered to be statistically significant and **p < 0.01.

## Results

### The miR-143 was strongly expressed in osteoblast cells and CD31^hi^EMCN^hi^ endothelial cells

**Fig. 1:**
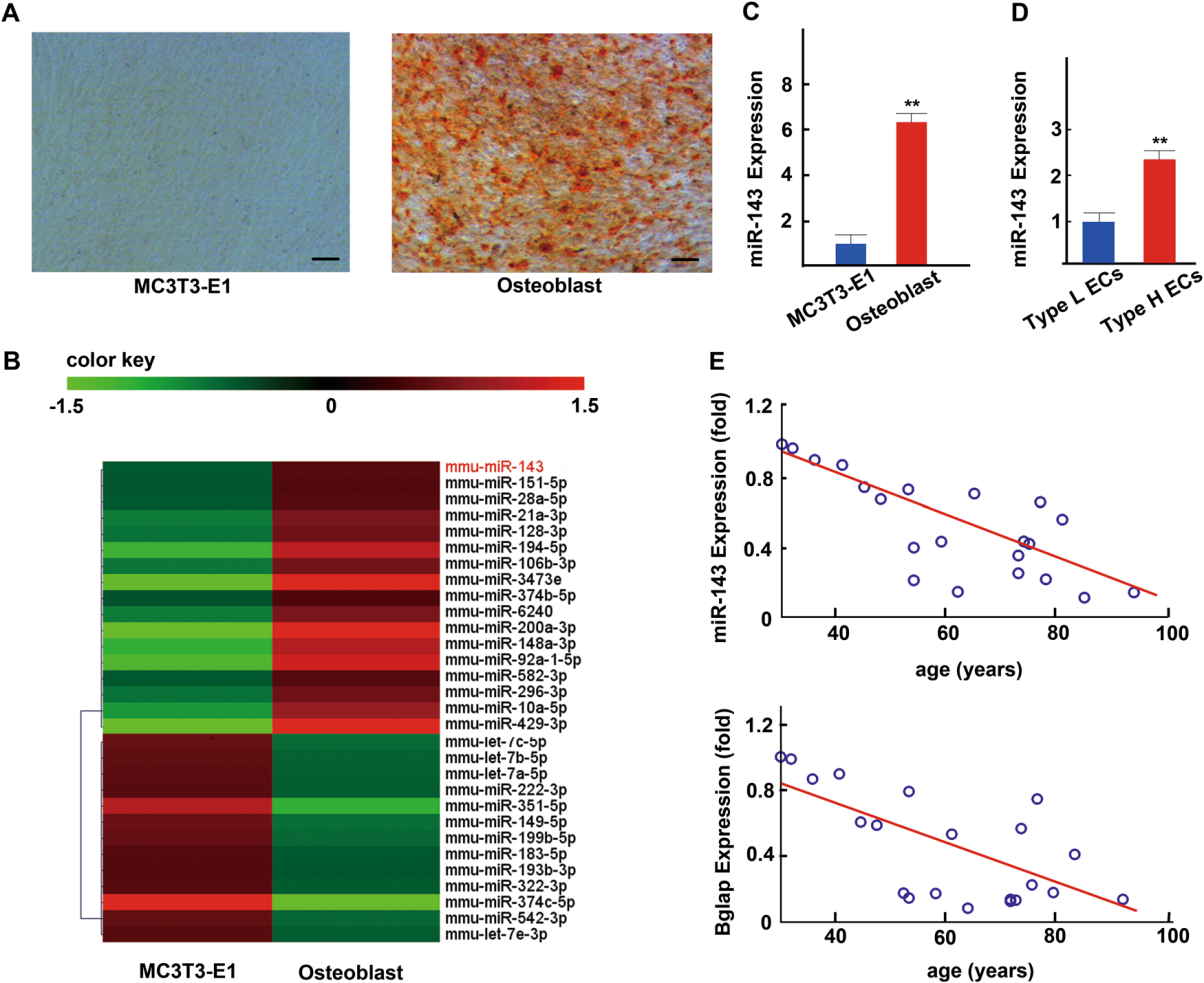
The miR-143 was strongly expressed in osteoblast cells and CD31^hi^EMCN^hi^ endothelial cells. **a** Representative images of Alizarin red S staining of matrix mineralization in MC3T3-E1 cells (Day 0) and osteoblast cultured in osteogenesis induction medium at day 21. Scale bar: 100 μm. **b** miRNA sequencing results of dysregulated miRNAs in MC3T3-E1 cells and osteoblasts. **c** qRT-PCR analysis of the levels of miR-143 expression in MC3T3-E1 cells and osteoblasts. **d** qRT-PCR analysis of the levels of miR-143 expression in CD31^hi^EMCN^hi^ and CD31^lo^EMCN^lo^ endothelial cells (Type-H ECs and Type-L ECs, respectively). **e** Age-associated changes of miR-143 and osteogenic biomarker BGLAP levels in 21 patients with femoral fractures. Data are reported as the mean ± SD. *p < 0.05, **p < 0.01.

To determine the role of miRNAs in osteoblast differentiation, pre-osteoblast MC3T3-E1 cells and MC3T3-E1 cells cultured in osteogenic differentiation medium for 21 days were screened for dysregulated miRNAs by performing miRNA sequencing analysis. Among the dysregulated miRNAs, miR-143 expression was six times greater in osteoblast than in MC3T3-E1 cells (Fig. 1a, b). Furthermore, the miR-143 levels of MC3T3-E1 was increased during osteogenic induction (Supplementary Fig. S1). Notably, the expression of miR-143 is higher in CD31^hi^EMCN^hi^ endothelial cells (Type-H ECs) than in CD31^lo^EMCN^lo^ endothelial cells (Type-L ECs)^19^. The increased expression level of miR-143 was further confirmed by quantitative real-time PCR (qRT-PCR) (Fig. 1c, d). Moreover, the miR-143 expression levels in the serum of patients with femoral fractures were negatively correlated with age and were positively correlated with the bone formation marker gene BGLAP (osteocalcin) expression levels (Fig. 1e). Thus, these data suggest that miR-143 levels were correlated with osteogenesis and angiogenesis.

### Overexpression of miR-143 promoted osteoblast differentiation

**Fig. 2:**
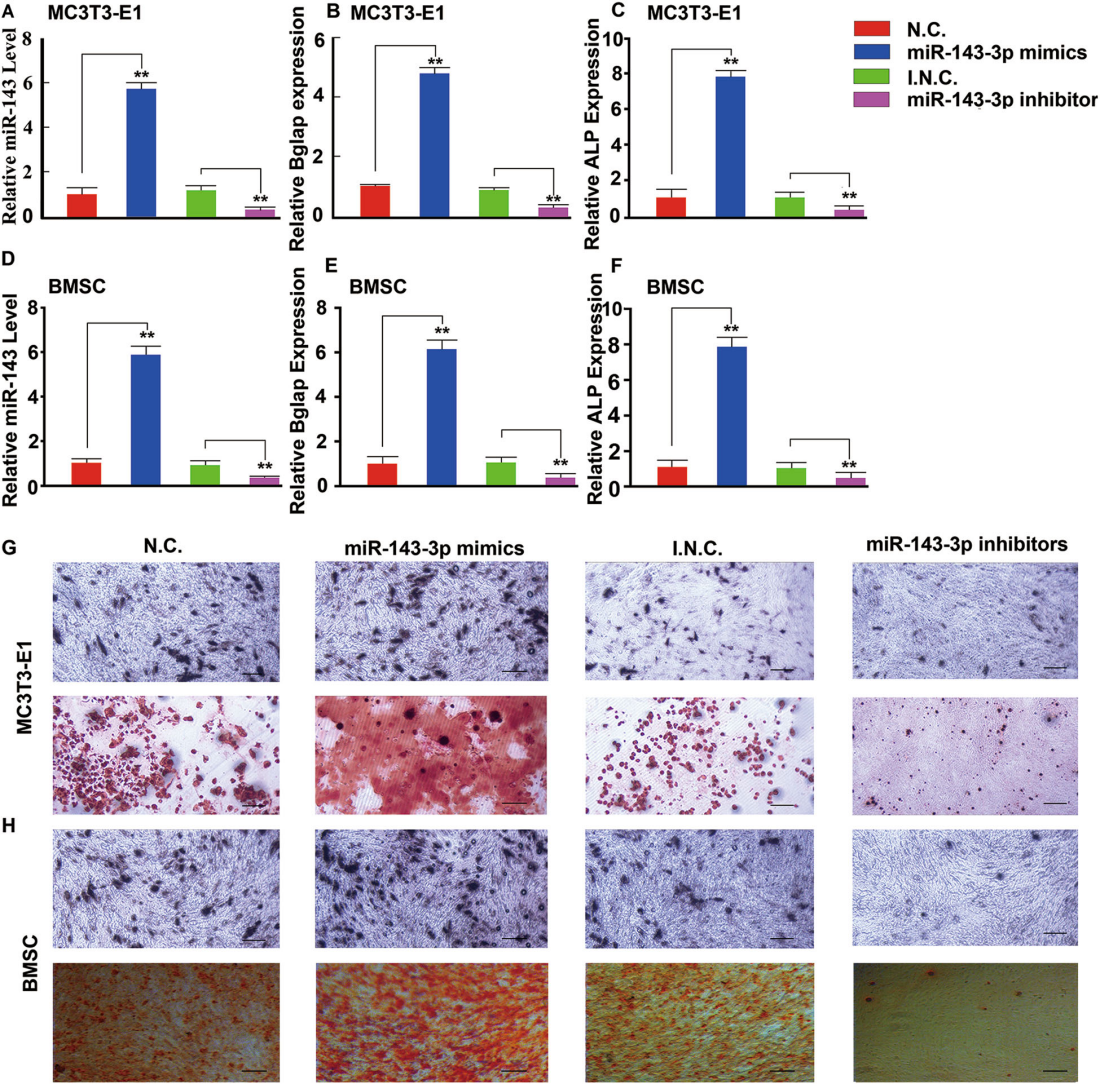
Overexpression of miR-143 promotes osteoblast formation. **a**, **d** qRT-PCR analysis of the relative levels of miR-143 expression in MC3T3-E1 cells and BMSCs transfected with miR-143 mimics, miR-143 inhibitors, or their respective negative controls at day 21. **b**, **c** qRT-PCR analysis of the relative levels of BGLAP (**b**) and Alp (**c**) mRNA expression in MC3T3-E1 cells at day 21. **e**, **f** qRT-PCR analysis of the relative levels of BGLAP (**e**) and Alp (**f**) mRNA expression in BMSCs at day 21. **g** Representative images of ALP (top) and Alizarin red S staining (bottom) in MC3T3-E1 cells transfected with miR-143 mimics, miR-143 inhibitors or their respective negative controls in osteogenic differentiation medium for 21 days. Scale bar: 100 μm. **h** Representative images of ALP (top) and Alizarin red S staining (bottom) in BMSCs transfected with miR-143 mimics, miR-143 inhibitors, or their respective negative controls in osteogenic differentiation medium for 21 days. Scale bar: 100 μm. Data are reported as the mean ± SD. *p < 0.05, **p < 0.01. Data are representative of three independent experiments.

To investigate osteogenesis regulated by miR-143 in vitro, we transfected mouse pre-osteoblast MC3T3-E1 cells with miR-143 mimics or inhibitors. The miR-143 expression levels were significantly upregulated by miR-143 mimic transfection and markedly inhibited by miR-143 inhibitor transfection (Fig. 2a). Furthermore, Bglap and Alp mRNA expression levels were upregulated by miR-143 mimics and downregulated by miR-143 inhibitors compared with those of their respective negative controls at day 21 (Fig. 2b–c and Supplementary Fig. S2A, B). Moreover, we also transfected primary BMSCs with miR-143 mimics and inhibitors in vitro, and found that BGLAP and Alp mRNA expression levels were upregulated by miR-143 mimics and downregulated by miR-143 inhibitors at day 21 (Fig. 2d–f and Supplementary Fig. S2A, B). Consistent with the results of BGLAP and Alp expression, we found that miR-143 mimics enhanced Alp staining, whereas miR-143 inhibitors weakened Alp staining (Fig. 2g, h). In addition, we found increased mineral deposition in miR-143 mimic-transfected cells and decreased mineral deposition in miR-143 inhibitor-transfected cells, compared with their negative control both in MC3T3-E1 cells and primary BMSCs (Fig. 2g, h). Overall, we concluded that osteoblast differentiation was promoted by miR-143 in vitro.

### MiR-143-mediated pro-angiogenic effects on BMECs

**Fig. 3:**
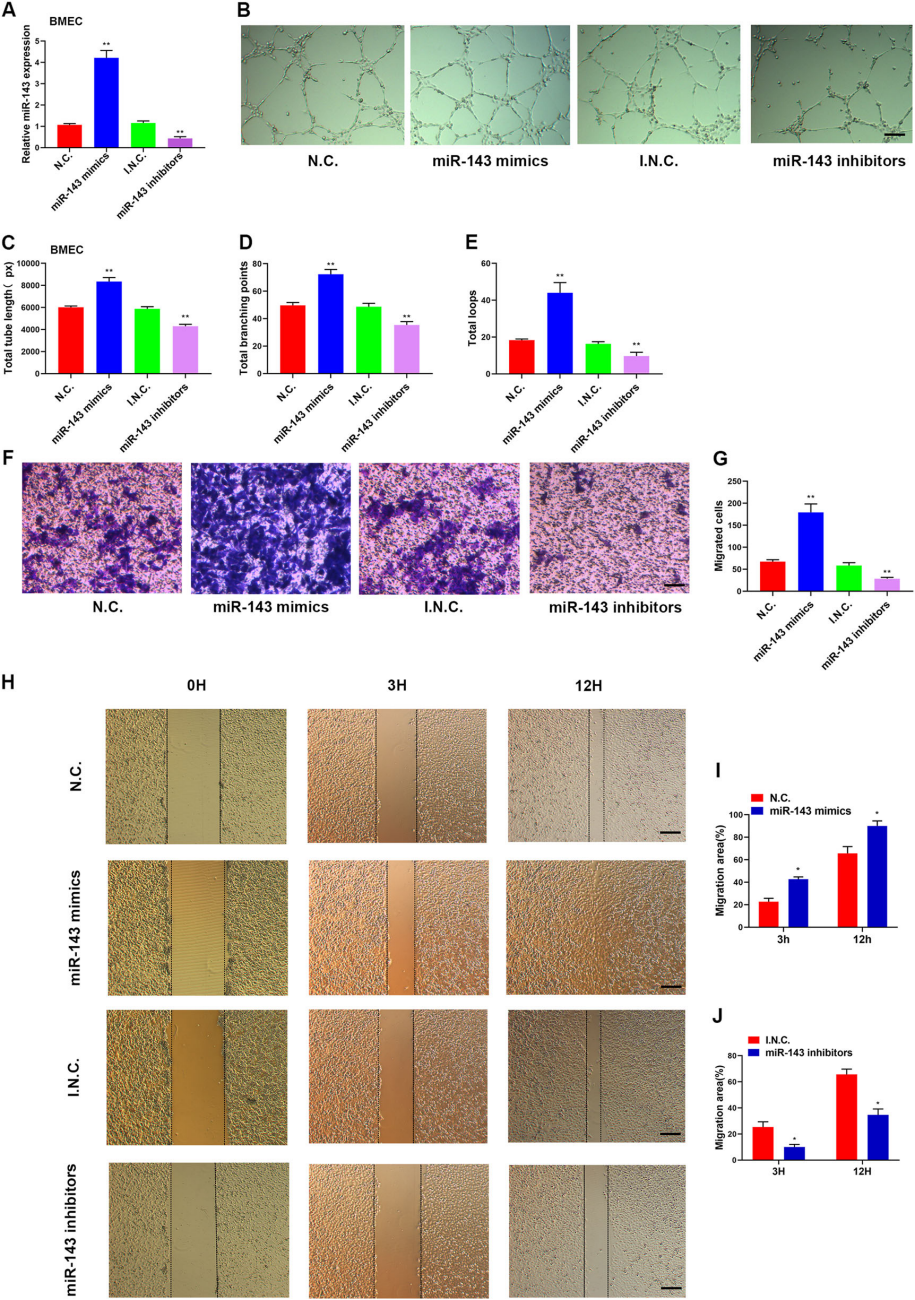
MiR-143 promotes angiogenic effects on BMECs. **a** qRT-PCR analysis of the relative levels of miR-143 expression in BMECs transfected with miR-143 mimics, miR-143 inhibitors, or their respective negative controls after 48 h. **b** Representative images of the tube formation assay on Matrigel in BMECs treated with miR-143 mimics, miR-143 inhibitors, or their respective negative controls 48 h later. Scale bar: 100 μm. **c**–**e** Quantitative analyses of the total tube length, total branching points, and total loops in **b**. *n* = 3 per group. Transwell assays (**f**, **g**) and scratch wound-healing assay (**h**–**j**) revealed that miR-143 promoted the migration of BMSCs. Scale bar: 100 μm. *N* = 3 per group. *P < 0.05, **P < 0.01.

To determine the role of miR-143 in endothelial cells in vitro, we transfected mouse BMECs with miR-143 mimics or inhibitors. The miR-143 expression levels were significantly increased by miR-143 mimic transfection and markedly inhibited by miR-143 inhibitor transfection (Fig. 3a). Furthermore, BMECs treated with miR-143 mimics showed a higher number of vascular-like structures compared with negative control in the tube formation assay on Matrigel, whereas miR-143 inhibitors showed fewer number of vascular-like structures (Fig. 3b). Quantitative experiments revealed that the total tube length, total branching points and total loops were upregulated by miR-143 mimics and downregulated by miR-143 inhibitors (Fig. 3c–e). Moreover, transwell assay (Fig. 3f, g) and scratch wound-healing assay (Fig. 3h–j) showed that miR-143 promoted the migration of BMSCs. In summary, these findings indicate that miR-143 promotes the angiogenic effects on BMECs.

### CD31^hi^Emcn^hi^ vessels and osteoblast differentiation were significantly inhibited in miR-143-knockout mice

**Fig. 4:**
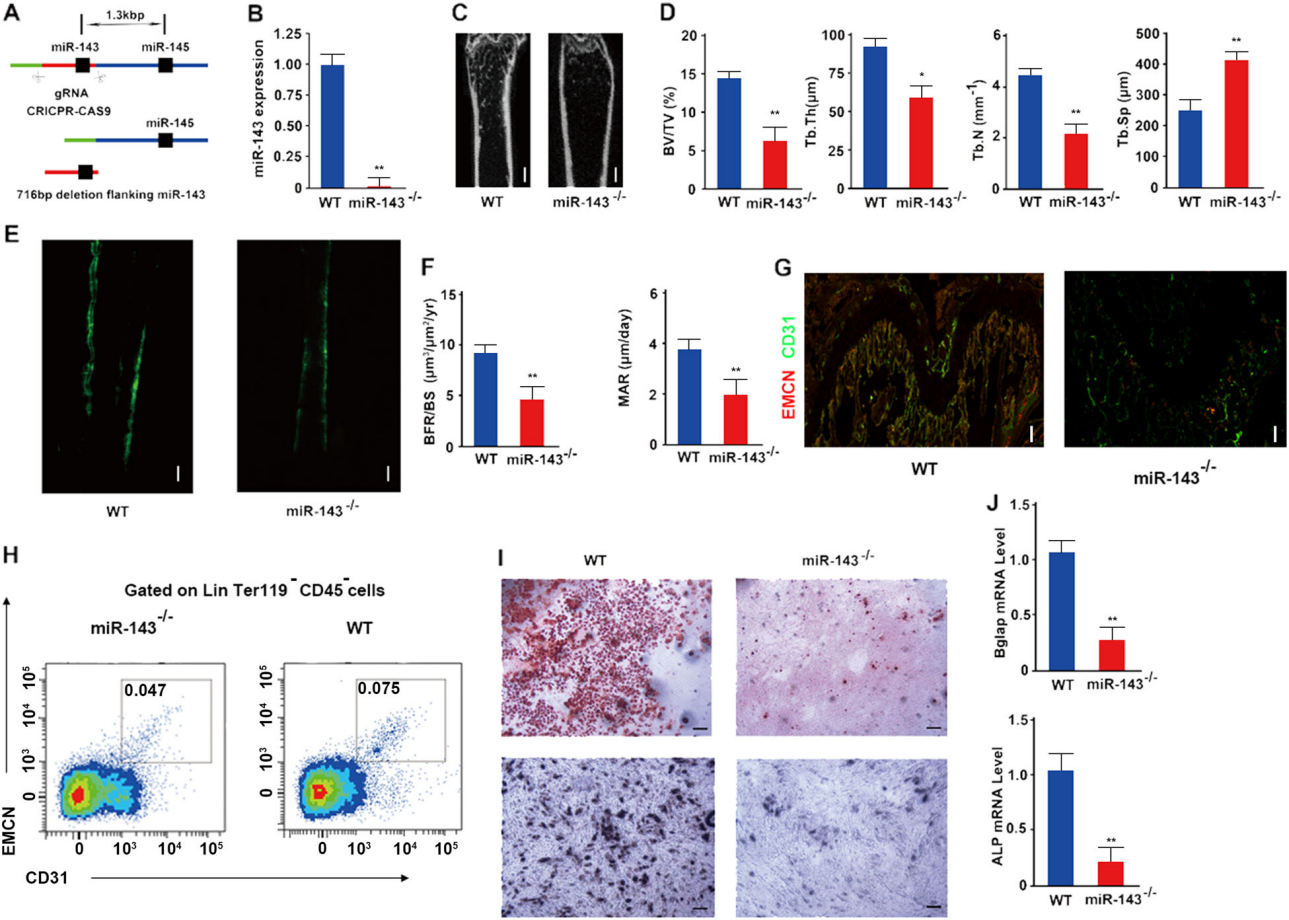
CD31^hi^Emcn^hi^ vessels and bone formation was significantly inhibited in miR-143-knockout mice. **a** A schematic diagram of miR-143-knockout mice constructed by CRISPR/Cas9 technology. **b** qRT-PCR analysis of the relative expression levels of miR-143 in miR-143 knockout mice and WT controls (*n* = 8 per group). **c** Representative microcomputed tomography images in the femora from WT mice and miR-143-knockout male mice (*n* = 8 per group). Scale bar: 100 μm. **d** Quantitative microcomputed tomography analysis of trabecular bone microarchitecture in femora from WT mice and miR-143-knockout male mice. BV/TV, trabecular bone volume per tissue volume; Tb. Th, trabecular thickness; Tb. Sp, trabecular separation; Tb. N, trabecular number (*n* = 8 per group). **e**, **f** Representative images of calcein double labeling of trabecular bone (**e**) with the quantification of the BFR per bone surface (BFR/BS) (**f** left) and the mineral apposition rate (MAR) (**f** right) (*n* = 8 per group). Scale bar: 100 μm. **g** Representative images of CD31 (green), Emcn (red) immunostaining. Scale bar: 100 μm. **h** FACS analysis dot plot of CD31^hi^Emcn^hi^ endothelial cells. **i** Representative images of Alizarin red S staining (top) and ALP staining (top) in BMSCs derived from WT and miR-143-knockout male mice cultured in osteogenic differentiation medium for 21 days. Scale bar: 50 μm. **j** qRT-PCR analysis of the relative levels of BGLAP (**h** left) and Alp (**h** right) mRNA expression in BMSCs derived from WT mice and miR-143-knockout male mice (*n* = 8 per group). Data are reported as the mean ± SD. *p < 0.05, **p < 0.01. Data are representative of three independent experiments.

To investigate the role of miR-143 in vivo, we generated miR-143-knockout (miR-143^−/−^) mice via CRISPR/Cas9 technology to confirm whether the depletion of miR-143 in vivo leads to bone loss (Fig. 4a). The knockout efficacy of miR-143 was confirmed in BMSCs through qRT-PCR, northern blot analysis, and PCR analysis (Fig. 4b and Supplementary Fig. S3). Microcomputed tomography (μCT) showed significantly decreased trabecular bone volume, number, and thickness, and increased trabecular separation in miR-143-knockout mice compared with those in WT controls (Fig. 4c–d). Calcein double labeling also indicated that miR-143-knockout mice had significantly decreased bone formation rates (BFRs) and decreased mineral apposition rates compared with those in WT controls (Fig. 4e–f). Furthermore, we found that CD31 and EMCN double-positive endothelium in the bone was significantly decreased in miR-143-knockout mice compared with their WT controls (Fig. 4g). Flow cytometry also confirmed fewer amounts of CD31^hi^Emcn^hi^ endothelial cells in bone marrow of miR-143^−/−^ mice (Fig. 4h). In vitro, BMSCs derived from miR-143-knockout mice showed decreased osteogenesis and decreased mineral deposition relative to the WT controls (Fig. 4i). Furthermore, BGLAP and ALP mRNA expression levels were downregulated in BMSCs derived from miR-143-knockout mice compared with those in WT controls (Fig. 4j). Therefore, all of these results suggest that CD31^hi^Emcn^hi^ vessels and osteoblast development were inhibited in miR-143-knockout mice.

### Angiogenic and osteogenic inhibitor HDAC7 was directly targeted by miR-143

**Fig. 5:**
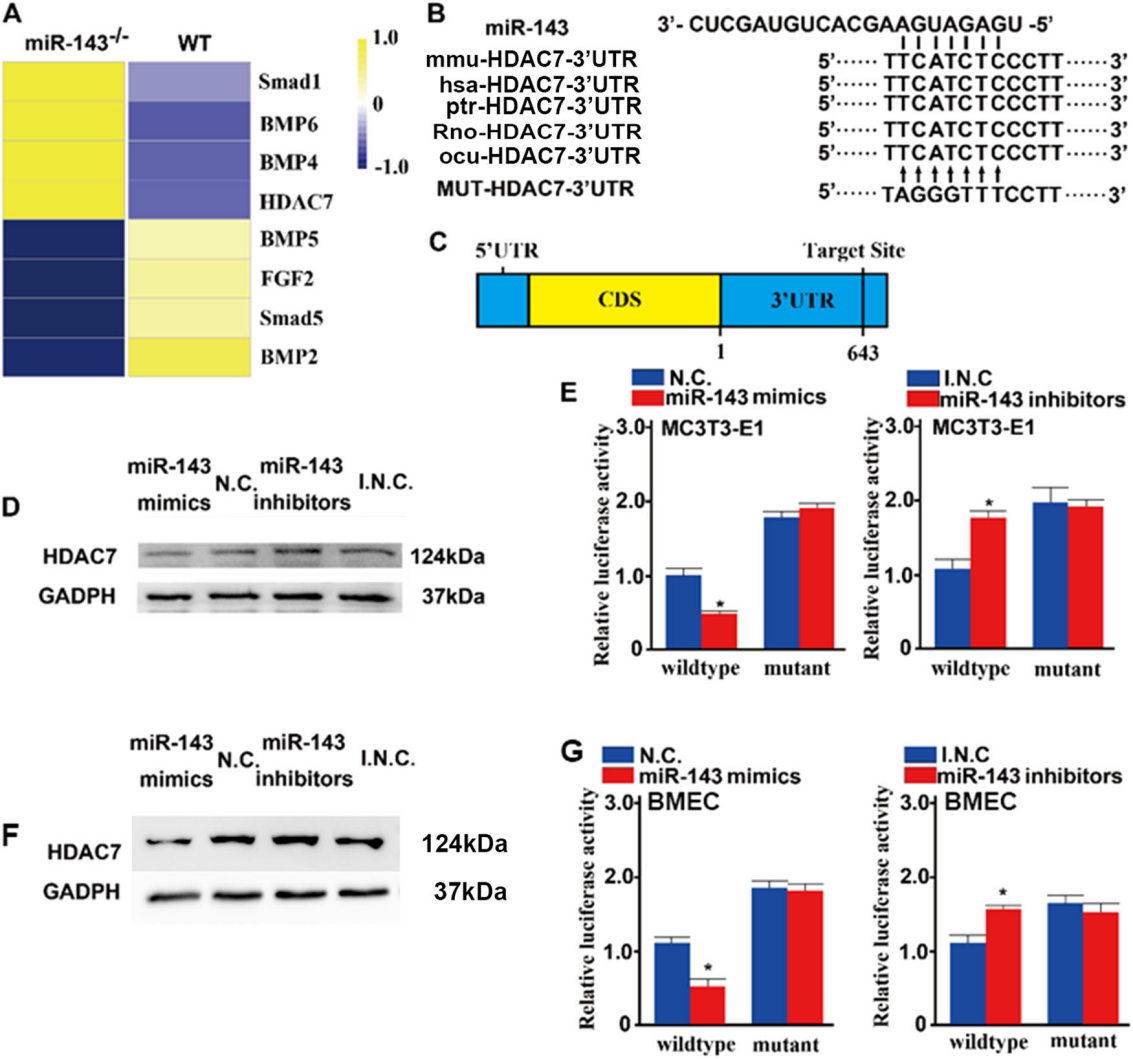
Angiogenic and osteogenic inhibitor HDAC7 was directly targeted by miR-143. **a** miRNA sequencing results of dysregulated mRNAs in BMSCs from WT and miR-143 knockout mice. **b** HDAC7 might be the target of miR-143. Sequences are shown for the miR-143 target sites in the 3′-UTR of HDAC7 mRNA and its mutant. **c** A schematic of miR-143 putative target sites in HDAC7. **d** Western blot analysis of HDAC7 and GADPH in MC3T3-E1 transfected with miR-143 mimics, miR-143 inhibitors, or their respective negative controls. **e** MC3T3-E1 cells were transfected with a luciferase reporter carrying WT or MUT-HDAC7 3′-UTR in miR-143 mimics (**e** left) and miR-143 (E, right) or their respective negative control for 48 h. The mean of control sample values was taken as “1” and the other samples were modulated according to it. **f** Western blot analysis of HDAC7 and GADPH in BMECs transfected with miR-143 mimics, miR-143 inhibitors, or their respective negative controls. **g** BMECs were transfected with a luciferase reporter carrying WT or MUT-HDAC7 3′-UTR in the miR-143 mimics (**f** left) and miR-143 (**f** right) or their respective negative controls for 48 h. The mean of control sample values was taken as “1” and the other samples were modulated according to it. Data are reported as the mean ± SD. *p < 0.05, **p < 0.01. Data are representative of three independent experiments.

The expression of mRNAs is regulated by miRNAs by binding to the 3′-UTRs of target mRNAs. To investigate the target mRNAs of miR-143 during osteoblast formation, BMSCs from miR-143-knockout mice and WT controls were screened for dysregulated mRNAs by performing mRNA-sequencing analysis (Fig. 5a). Combining the results of miR-143 target prediction in TargetScan and miRanda, HDAC7 was suggested to be the potential target of miR-143 (Fig. 5b, c). Furthermore, we have checked the miRNA-binding site in Targetscan and found that miR-143 binding site on HDAC7 3′-UTR is conserved among the species (Fig. 5b). Previously, HDAC7 has been reported to associate with Runx2-responsive promoter elements in osseous cells and inhibit osteoblast maturation in a deacetylation-independent manner^20^. Furthermore, HDAC7 inhibits endothelial cell growth through β-catenin, including CD31^hi^EMCN^hi^ type-H endothelial cells^21^. Thus, we focused on the interaction between HDAC7 and miR-143 in this study, and we found that overexpression of miR-143 suppressed endogenous levels of HDAC7 protein expression, while inhibition of miR-143 enhanced HDAC7 protein expression (Fig. 5d, f and Supplementary Fig. S4A, B).

To determine whether miR-143 can directly target HDAC7, luciferase reporter constructs containing the WT or mutated predicted miRNA-binding sites of HDAC7 (WT-HDAC7–3′-UTR and MUT-HDAC7–3′-UTR, respectively) were generated (Fig. 5b–c). We transfected WT-HDAC7–3′-UTR or MUT-HDAC7–3′-UTR with miR-143 mimics or negative controls into MC3T3-E1 cells and measured the effects of miR-143 on luciferase translation by the level of luciferase enzyme activity. miR-143 mimics inhibited the luciferase activity of the HDAC7 3′-UTR reporter gene, whereas MUT-HDAC7–3′-UTR prevented this inhibition (Fig. 5e). Furthermore, the luciferase reporter experiment of miR-143 inhibitors or the inhibitor negative controls showed that miR-143 increased the luciferase activity of the HDAC7 3′-UTR reporter gene, whereas MUT-HDAC7–3′-UTR prevented this inhibition (Fig. 5e). In addition, the luciferase reporter experiment with BMECs showed similar results compared with those with MC3T3-E1 cells (Fig. 5g). In conclusion, these results show that miR-143 directly targets the 3′-UTR of HDAC7 and inhibits HDAC7 expression in both osteoblasts and endothelial cells.

### Inhibition of HDAC7 rescued the function of miR-143 deficiency in mice

**Fig. 6:**
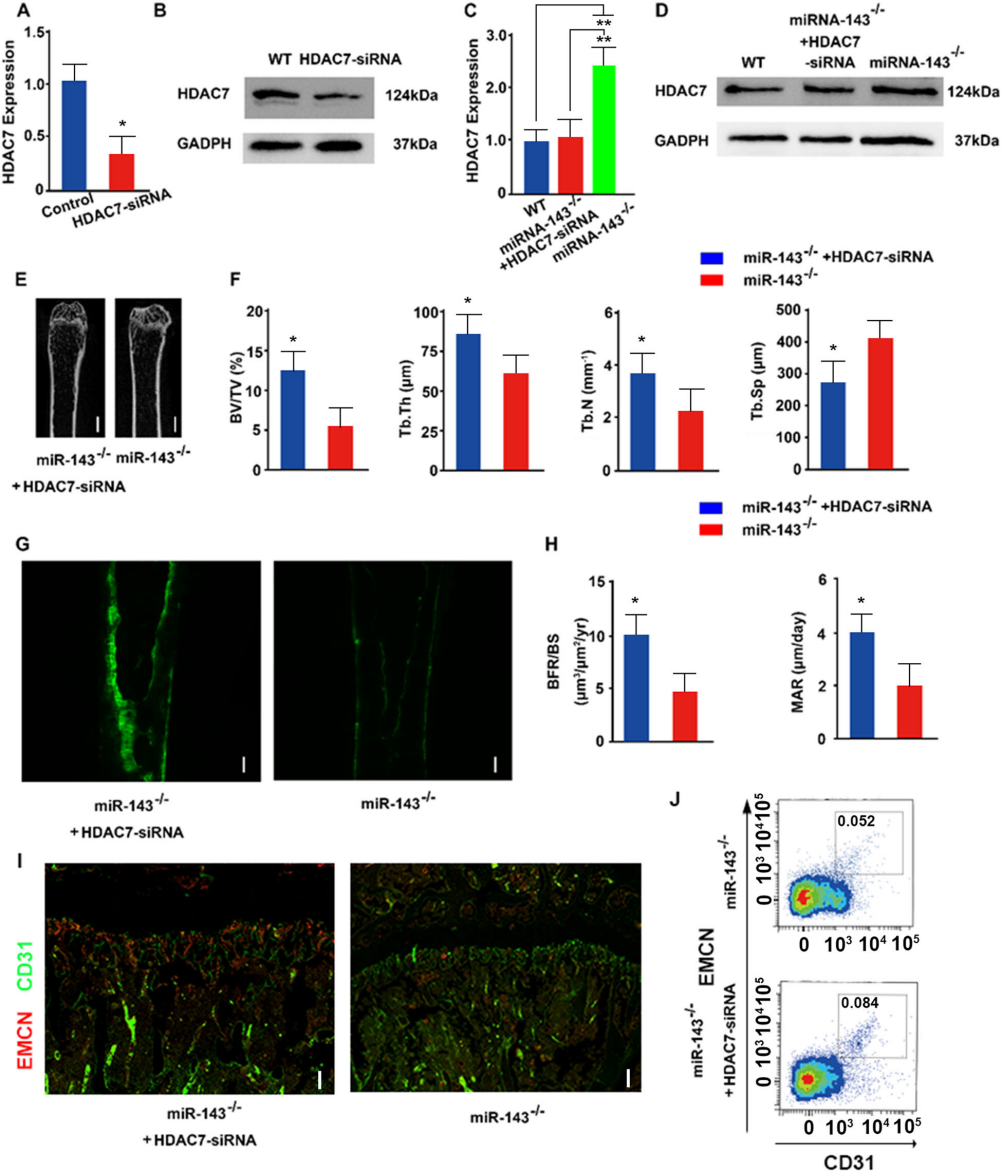
Inhibition of HDAC7 rescues the function of miR-143 deficiency in mice. qRT-PCR analysis (**a**) of the relative expression levels of HDAC7 and western blot analysis (**b**). qRT-PCR analysis (**c**) of the relative levels of HDAC7 and Western blot analysis (**d**) of HDAC7 and GADPH in BMSCs derived from WT, miR-143 knockout mice transfected with HDAC7-siRNA, and miR-143-knockout male mice (*n* = 8 per group). Microcomputed tomography images (**e**) of trabecular bone microarchitecture in femora and quantitative microcomputed tomography analysis (**f**) of trabecular bone microarchitecture from miR-143-knockout mice injected with cholesterol-conjugated control RNA or HDAC7-siRNA in the tail vein (*n* = 8 per group). Scale bar: 100 μm. **g**, **h** Representative images of calcein double labeling of trabecular bone (**g**) with quantification of the BFR per bone surface (BFR/BS) (**h** left) and the mineral apposition rate (MAR) (**h** right) (*n* = 8 per group). Scale bar: 100 μm. **i** Representative images of CD31 (green), Emcn (red) immunostaining from miR-143-knockout mice injected with cholesterol-conjugated control RNA or HDAC7-siRNA in the tail vein. Scale bar: 100 μm. **j** FACS analysis dot plot of CD31^hi^Emcn^hi^ endothelial cells from miR-143-knockout mice injected with cholesterol-conjugated control RNA or HDAC7-siRNA in the tail vein. Data are reported as the mean ± SD. *p < 0.05, **p < 0.01. Data are representative of three independent experiments.

To confirm the role of HDAC7 in bone formation in vitro, we transfected BMSCs with HDAC7-siRNA to silence HDAC7 expression and confirmed its efficacy (Fig. 6a, b and Supplementary Fig. S4C). Furthermore, we transfected BMSCs from miR-143-knockout mice with HDAC7-siRNA and determined that HDAC7 expression was inhibited by HDAC7 knockdown (Fig. 6c, d and Supplementary Fig. S4D). Then, to investigate the role of HDAC7 in vivo, we administered cholesterol-conjugated control RNA or HDAC7-siRNA into miR-143-knockout mice to determine whether HDAC7 inhibition could rescue the bone loss mediated by miR-143 deficiency in vivo^22^. μCT analysis showed significantly increased trabecular bone volume, number, and thickness, and decreased trabecular separation in miR-143-knockout mice transfected with cholesterol-conjugated HDAC7-siRNA compared with those in miR-143-knockout mice (Fig. 6e, f). Calcein double labeling also indicated that miR-143-knockout mice transfected with cholesterol-conjugated HDAC7-siRNA had significantly decreased BFRs compared with those in miR-143 knockout mice (Fig. 6g, h). Furthermore, miR-143^−/−^ mice transfected with cholesterol-conjugated HDAC7-siRNA showed increased number of CD31^hi^Emcn^hi^ endothelial cells relative to those miR-143^−/−^ mice by immunofluorescence and flow cytometric analysis (Fig. 6i, j). Bglap and Alp expression levels were decreased by miR-143 deficiency and rescued by knockdown of HDAC7 in vivo (Supplementary Fig. S5A, B), which confirming the roles of miR-143 and HDAC7 in osteogenesis. Overall, these results demonstrated that miR-143 promoted angiogenesis coupling with osteoblast differentiation by targeting HDAC7.

### Administration of miR-143 promotes angiogenesis and osteogenesis in vivo

**Fig. 7:**
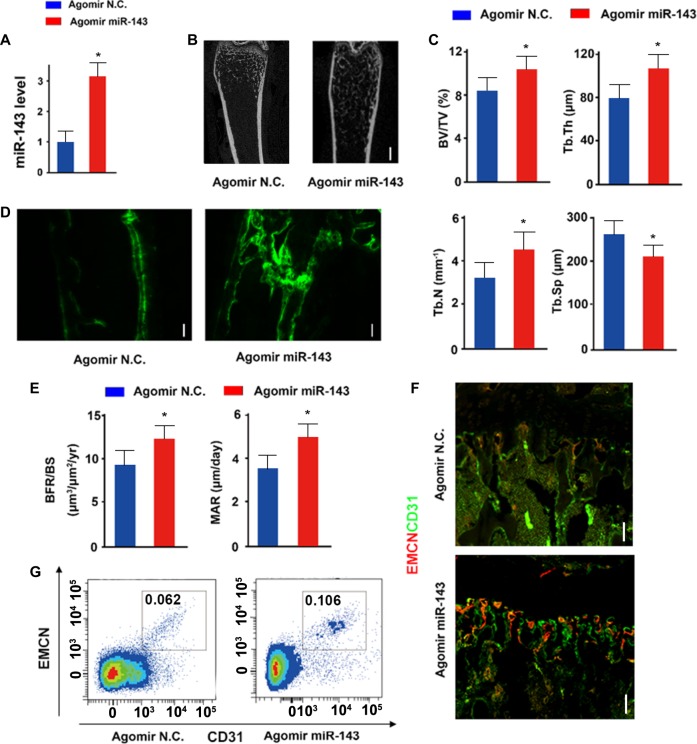
Administration of miR-143 promotes angiogenesis and osteogenesis in vivo. **a** qRT-PCR analysis of the levels of miR-143 expression in BMSCs of mice injected with agomiR-143 or agomiR-NC. NC indicates the negative control (*n* = 8 per group). Microcomputed tomography images (**b**) of trabecular bone microarchitecture in femora and quantitative microcomputed tomography analysis (**c**) of trabecular bone microarchitecture from miR-143-knockout male mice treated with agomiR-143 and agomiR-NC (*n* = 8 per group). Scale bar: 100 μm. **d**, **e** Representative images of calcein double labeling of trabecular bone (**d**) with the quantification of the BFR per bone surface (BFR/BS) (**e** left) and the mineral apposition rate (MAR) (**e** right) (*n* = 8 per group). Scale bar: 100 μm. **f** Representative images of CD31 (green), Emcn (red) immunostaining from mice injected with agomiR-143 or agomiR-NC. Scale bar: 100 μm. **g** FACS analysis dot plot of CD31^hi^Emcn^hi^ endothelial cells from mice injected with agomiR-143 or agomiR-NC. Data are reported as the mean ± SD. *p < 0.05, **p < 0.01. Data are representative of three independent experiments.

To investigate the therapeutic potential of miR-143 on age-related osteoporosis, agomiR-143 or agomiR-NC (negative control) was injected into the tail vein of 12-month-old female mice once per week for 12 weeks. Mice injected with agomiR-143 significantly increased the levels of miR-143 expression in BMSCs compared with those in the mice injected with agomiR-NC (Fig. 7a). Furthermore, we found that HDAC7 expression was inhibited in BMSCs transfected with agomiR-143 by western blot analysis (Supplementary Fig. S6). Mice treated with agomiR-143 showed increased trabecular bone volume, number, and thickness, and decreased trabecular separation relative to the agomiR-NC controls (Fig. 7b, c). Furthermore, Calcein double labeling showed that the BFR was increased in mice injected with agomiR-143 compared to that in the agomiR-NC controls (Fig. 7d, e). The amounts of CD31^hi^EMCN^hi^ endothelial cells was significantly increased in mice treated with agomiR-143 than in mice treated with the agomiR-NC (Fig. 7f). Fluorescence-activated cell sorting (FACS) analysis was also consistent with the result of immunofluorescence (Fig. 7g). In conclusion, these results show that intravenous injection of agomiR-143 promoted bone formation and prevented bone loss on age-related osteoporosis.

## Discussion

In this study, we found that miR-143 was significantly increased in osteoblast cells and CD31^hi^EMCN^hi^ endothelial cells and was positively correlated with the bone formation marker genes BGLAP and Alp. Overexpression of miR-143 promoted osteoblast differentiation and mediated pro-angiogenic effects on BMECs. Furthermore, we generated miR-143-knockout mice and confirmed that bone formation was inhibited in miR-143-knockout mice in vivo. Moreover, we found that miR-143 promotes angiogenesis coupling with osteoblast differentiation by targeting HDAC7, and that inhibition of HDAC7 rescues bone formation inhibited by miR-143 deficiency in mice. In addition, agomiR-143 treatment in vivo ameliorated bone loss and age-related osteoporosis.

Previously, we reported that miR-143 was downregulated in osteosarcoma, and that miR-143 promotes apoptosis and suppresses tumorigenicity by targeting Bcl-2^23^. In addition, several miRNAs have been reported to be dysregulated in osteosarcoma^24^. As miR-422a inhibits the proliferation of osteosarcoma cells by targeting BCL2L2 and KRAS^25^, miR-148a promotes cancer cell growth in osteosarcoma by targeting phosphatase and tensin homolog (PTEN)^26^ and miR-133a inhibits osteosarcoma progression by targeting Bcl-xL and Mcl-1^27^. In this study, we confirmed that miR-143 is the regulator of bone formation; thus, miR-143 is involved in both osteoblast differentiation and carcinogenesis. As a set of miRNAs is dysregulated in osteosarcoma, we hypothesized that these miRNAs might also have important roles in the regulation of bone formation. Our work might provoke interesting future works regarding the correlation between bone formation and osteosarcoma progression leading to new potential therapeutic targets.

The serum level of miR-143 was found to be decreased in aged patients and administration of miR-143 prevented bone loss in age-related osteoporosis. Previously, a set of miRNAs has been reported to be correlated with age and several of them have been reported to modulate osteoblast differentiation^28^. For example, the miR-188 expression level is positively correlated with age and regulates the age-related regulation of osteoblast and adipocyte differentiation^29^, and miR-29 is substantially upregulated in multiple tissues with increasing age and modulates Wnt signaling in human osteoblasts^30,31^. These miRNAs may also bear therapeutic potential in the targeted therapy of age-related osteoporosis, which requires further investigation.

HDACs are conserved enzymes that remove acetyl groups from lysine side chains in histones. Previously, many HDACs have been reported to regulate intramembranous and endochondral ossification as well as bone resorption. HDAC7 is a class IIa HDACs that plays crucial roles in bone formation by suppressing Runx2 activity and inhibiting osteoclastogenesis by reversing the RANKL-triggered beta-catenin switch^32,33^. HDAC inhibitors (HDIs) induce apoptosis through the death receptor pathway^34^ and they have been used as therapeutics for cancer. Moreover, the ability of HDIs to suppress human osteosarcomas, chondrosarcomas, and other primary bone tumors has been extensively reported through in vitro studies^35–41^. In our previous studies, several target genes were found to participate in the regulation of osteosarcoma, such as BCL-2, PTEN, BCL-xL, and KRAS. Several of these genes have been reported to regulate osteoblast formation, such as overexpression of BCL-xL in osteoblasts that inhibits osteoblast apoptosis^42^ and inhibition of PTEN that promotes the accumulation of the bone^43^. These results suggest that the genes involved in osteosarcoma might also regulate bone formation and this presumption may provide new insights into the molecular mechanisms of bone formation.

## Supplementary information

supplementary figure legends

Figure S1

Figure S2

Figure S3

Figure S4

Figure S5

Figure S6

## References

[1] Karsenty G (2003). The complexities of skeletal biology. Nature.

[2] Chang J (2009). Inhibition of osteoblastic bone formation by nuclear factor-kappaB. Nat. Med..

[3] Kusumbe AP, Adams RH (2014). Osteoclast progenitors promote bone vascularization and osteogenesis. Nat. Med..

[4] Tang Y (2009). TGF-beta1-induced migration of bone mesenchymal stem cells couples bone resorption with formation. Nat. Med..

[5] Ikebuchi Y (2018). Coupling of bone resorption and formation by RANKL reverse signalling. Nature.

[6] Koga T (2005). NFAT and Osterix cooperatively regulate bone formation. Nat. Med.

[7] Nakashima K (2002). The novel zinc finger-containing transcription factor osterix is required for osteoblast differentiation and bone formation. Cell.

[8] Kusumbe AP, Ramasamy SK, Adams RH (2014). Coupling of angiogenesis and osteogenesis by a specific vessel subtype in bone. Nature.

[9] Ramasamy SK, Kusumbe AP, Wang L, Adams RH (2014). Endothelial Notch activity promotes angiogenesis and osteogenesis in bone. Nature.

[10] Bartel DP (2004). MicroRNAs: genomics, biogenesis, mechanism, and function. Cell.

[11] Kim VN, Han J, Siomi MC (2009). Biogenesis of small RNAs in animals. Nat. Rev. Mol. Cell Biol..

[12] Hou J (2011). Identification of miRNomes in human liver and hepatocellular carcinoma reveals miR-199a/b-3p as therapeutic target for hepatocellular carcinoma. Cancer Cell..

[13] Hou J (2009). MicroRNA-146a feedback inhibits RIG-I-dependent type I IFN production in macrophages by targeting TRAF6, IRAK1, and IRAK2. J. Immunol..

[14] Bradley EW (2015). Histone deacetylases in bone development and skeletal disorders. Physiol. Rev..

[15] Højfeldt JW, Agger K, Helin K (2013). Histone lysine demethylases as targets for anticancer therapy. Nat. Rev. Drug Discov..

[16] Hull EE, Montgomery MR, Leyva KJ (2016). HDAC inhibitors as epigenetic regulators of the immune system: impacts on cancer therapy and inflammatory diseases. Biomed. Res. Int..

[17] Kang JS, Alliston T, Delston R, Derynck R (2005). Repression of Runx2 function by TGF-beta through recruitment of class II histone deacetylases by Smad3. EMBO J..

[18] Huang J (2018). Harmine enhances type H vessel formation and prevents bone loss in ovariectomized mice. Theranostics.

[19] Yang M (2017). MiR-497~195 cluster regulates angiogenesis during coupling with osteogenesis by maintaining endothelial Notch and HIF-1a activity. Nat. Commun..

[20] Jensen ED, Schroeder TM, Bailey J, Gopalakrishnan R, Westendorf JJ (2008). Histone deacetylase 7 associates with Runx2 and represses its activity during osteoblast maturation in a deacetylation-independent manner. J. Bone Min. Res..

[21] Margariti A (2010). Histone deacetylase 7 controls endothelial cell growth through modulation of β-catenin. Circ. Res..

[22] Hou J (2014). Hepatic RIG-I predicts survival and interferon-a therapeutic response in hepatocellular carcinoma. Cancer Cell.

[23] Zhang H (2010). microRNA-143, down-regulated in osteosarcoma, promotes apoptosis and suppresses tumorigenicity by targeting Bcl-2. Oncol. Rep..

[24] De Vito C (2012). A TARBP2-dependent miRNA expression profile underlies cancer stem cell properties and provides candidate therapeutic reagents in Ewing sarcoma. Cancer Cell.

[25] Zhang, H. et al. miR-422a inhibits osteosarcoma proliferation by targeting BCL2L2 and KRAS. *Biosci. Rep*. **38**, BSR20170339 (2018). 10.1042/BSR20170339 PMC5861329 29358307

[26] Zhang H (2016). Increased expression of microRNA-148a in osteosarcoma promotes cancer cell growth by targeting PTEN. Oncol. Lett..

[27] Ji F (2013). MicroRNA-133a, downregulated in osteosarcoma, suppresses proliferation and promotes apoptosis by targeting Bcl-xL and Mcl-1. Bone.

[28] Sun K, Lai EC (2014). Adult-specific functions of animal microRNAs. Nat. Rev. Genet..

[29] Li CJ (2015). MicroRNA-188 regulates age-related switch between osteoblast and adipocyte differentiation. J. Clin. Invest..

[30] Ugalde AP (2011). Aging and chronic DNA damage response activate a regulatory pathway involving miR-29 and p53. EMBO J..

[31] Kapinas K, Kessler C, Ricks T, Gronowicz G, Delany AM (2010). miR-29 modulates Wnt signaling in human osteoblasts through a positive feedback loop. J. Biol. Chem..

[32] Bradley EW, Carpio LR, Olson EN, Westendorf JJ (2015). Histone deacetylase 7 (Hdac7) suppresses chondrocyte proliferation and beta-catenin activity during endochondral ossification. J. Biol. Chem..

[33] Jin Z, Wei W, Dechow PC, Wan Y (2013). HDAC7 inhibits osteoclastogenesis by reversing RANKL-triggered beta-catenin switch. Mol. Endocrinol..

[34] Insinga A (2005). Inhibitors of histone deacetylases induce tumor-selective apoptosis through activation of the death receptor pathway. Nat. Med..

[35] Capobianco E (2014). Separate and combined effects of DNMT and HDAC inhibitors in treating human multi-drug resistant osteosarcoma HosDXR150 cell line. PLoS ONE.

[36] Thayanithy V (2012). Combinatorial treatment of DNA and chromatin-modifying drugs cause cell death in human and canine osteosarcoma cell lines. PLoS ONE.

[37] Tonak M (2014). HDAC inhibitor-loaded bone cement for advanced local treatment of osteosarcoma and chondrosarcoma. Anticancer Res..

[38] Wittenburg LA, Bisson L, Rose BJ, Korch C, Thamm DH (2011). The histone deacetylase inhibitor valproic acid sensitizes human and canine osteosarcoma to doxorubicin. Cancer Chemother. Pharm..

[39] Wittenburg LA, Ptitsyn AA, Thamm DH (2012). A systems biology approach to identify molecular pathways altered by HDAC inhibition in osteosarcoma. J. Cell Biochem..

[40] Yamamoto S (2008). Suberoylanilide hydroxamic acid (SAHA) induces apoptosis or autophagy-associated cell death in chondrosarcoma cell lines. Anticancer Res..

[41] Cassier PA (2014). Outcome of patients with sarcoma and other mesenchymal tumours participating in phase I trials: a subset analysis of a European Phase I database. Ann. Oncol..

[42] Moriishi T (2016). Overexpression of BCLXL in osteoblasts inhibits osteoblast apoptosis and increases bone volume and strength. J. Bone Min. Res..

[43] Liu X (2007). Lifelong accumulation of bone in mice lacking Pten in osteoblasts. PNAS.

