# Supplementary material for: miR-143 promotes angiogenesis and osteoblast differentiation by targeting HDAC7
Source: Cell Death Dis. 2020 Mar 9;11(3):179. doi: 10.1038/s41419-020-2377-4 (PMC7062786; doi:10.1038/s41419-020-2377-4)
Supplement: Supplementary file 1 — supplementary figure legends [file 41419_2020_2377_MOESM1_ESM.docx]

**Supplementary Figure Legends**

Figure S1. RT-qPCR analysis of miR-143 expression of MC3T3-E1 cultured in osteogenic induction medium at day 0, 7, 14, and 21. Data are reported as the mean ± s.d. ** p < 0.01.

Figure S2. RT-qPCR analysis of miR-143 expression of MC3T3-E1 transfected with miR-143 mimics or N.C. (A) and inhibitors or I.N.C. (B) cultured in osteogenic induction medium at day 0, 7, 14, and 21. Data are reported as the mean ± s.d. ** p < 0.01.

Figure S3. (A). The Northern blot analysis of mmu-miR-143 and U6 snRNA in BMSCs derived from miR-143 knockout mice and their controls. (B). PCR analysis data of miR-143 knockout mice (-/-), together with their WT controls (+/+) and heterozygous (+/-). (C). RT-qPCR analysis of miR-145 expression of BMSCs derived from miR-143 knockout mice and their WT controls. Data are reported as the mean ± s.d.

Figure S4. The quantification analysis of the Western Blot by densitometric analysis in Figure 5D (A), Figure 5F (B), Figure 6B (C), Figure 6D (D). Data are reported as the mean ± s.d. ** p < 0.01.

Figure S5. qRT-PCR analysis of the relative expression levels of Bglap and Alp in BMSCs derived from WT, miR-143 knockout mice transfected with HDAC7-siRNA and miR-143 knockout mice. Data are reported as the mean ± s.d. ** p < 0.01.

Figure S6. Western blot analysis of HDAC7 and GADPH in BMSCs of mice injected with Agomir miR-143 or Agomir N.C..
