# Supplementary figures and images for: miR-143 promotes angiogenesis and osteoblast differentiation by targeting HDAC7
Source: Cell Death Dis. 2020 Mar 9;11(3):179. doi: 10.1038/s41419-020-2377-4 (PMC7062786; doi:10.1038/s41419-020-2377-4)

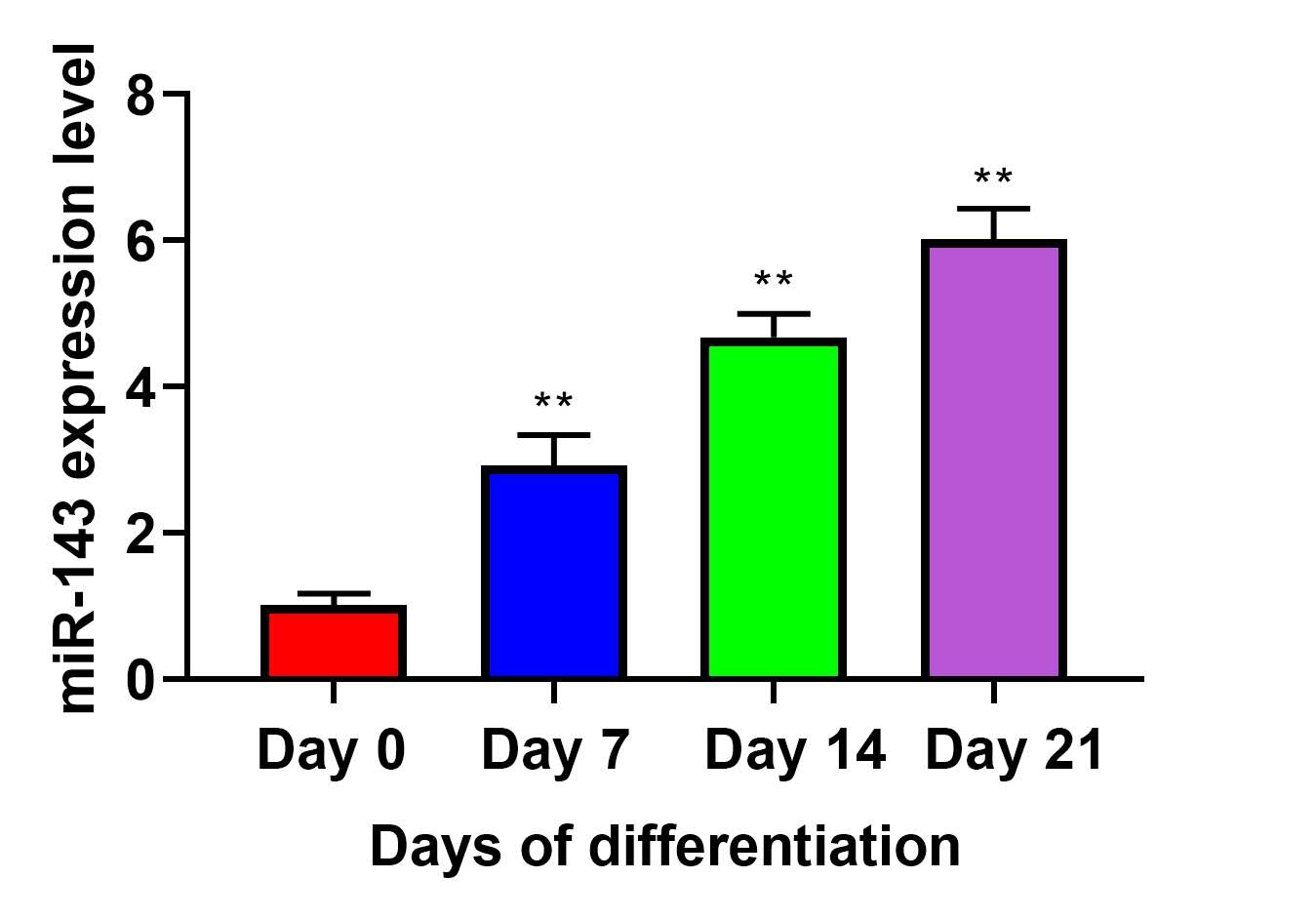

Supplement: Supplementary file 2 — Figure S1 [file 41419_2020_2377_MOESM2_ESM.tif]

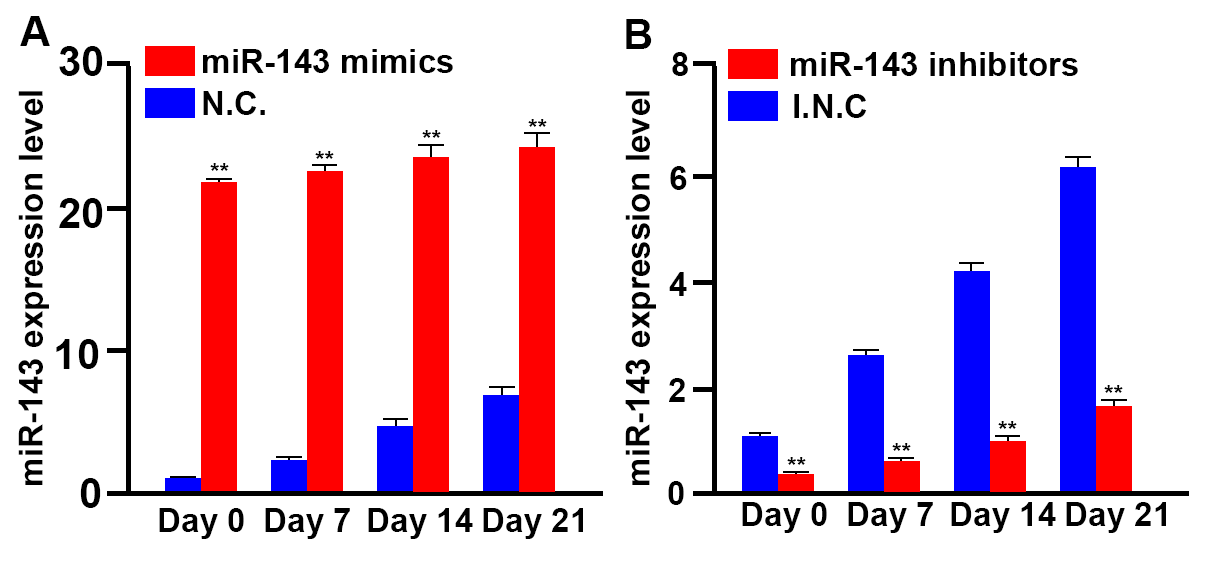

Supplement: Supplementary file 3 — Figure S2 [file 41419_2020_2377_MOESM3_ESM.tif]

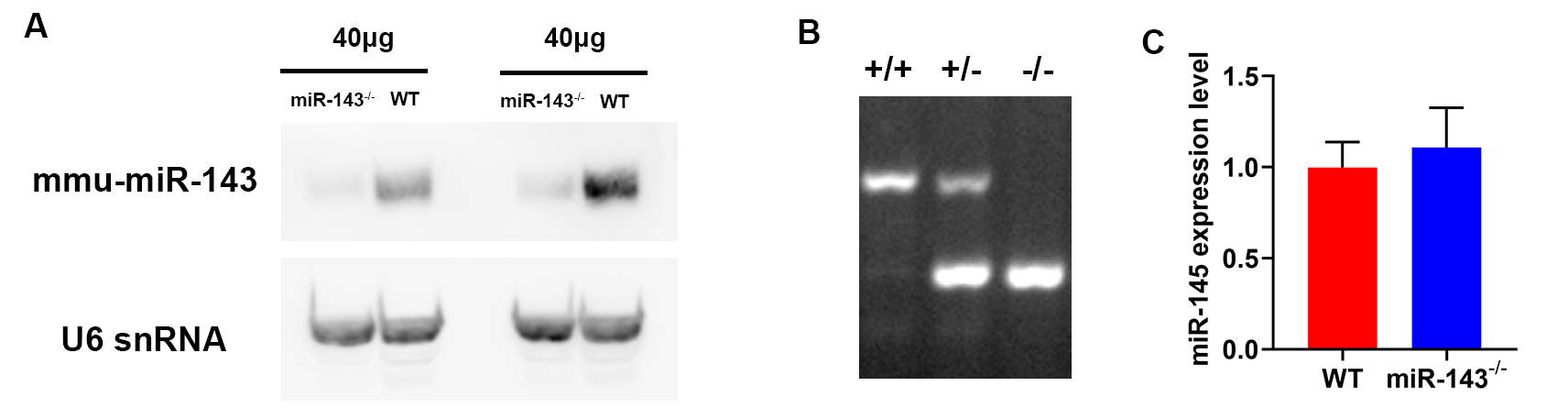

Supplement: Supplementary file 4 — Figure S3 [file 41419_2020_2377_MOESM4_ESM.tif]

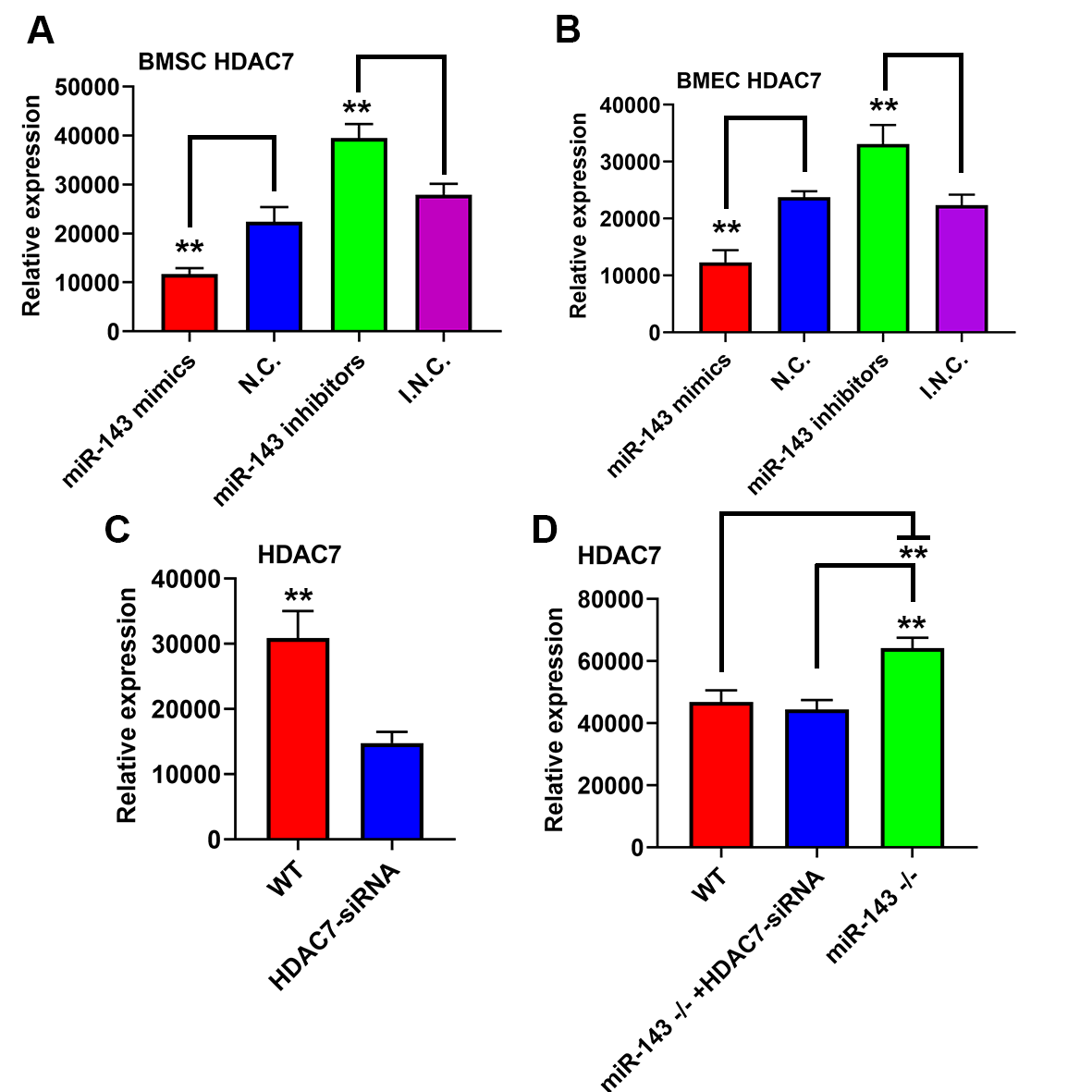

Supplement: Supplementary file 5 — Figure S4 [file 41419_2020_2377_MOESM5_ESM.tif]

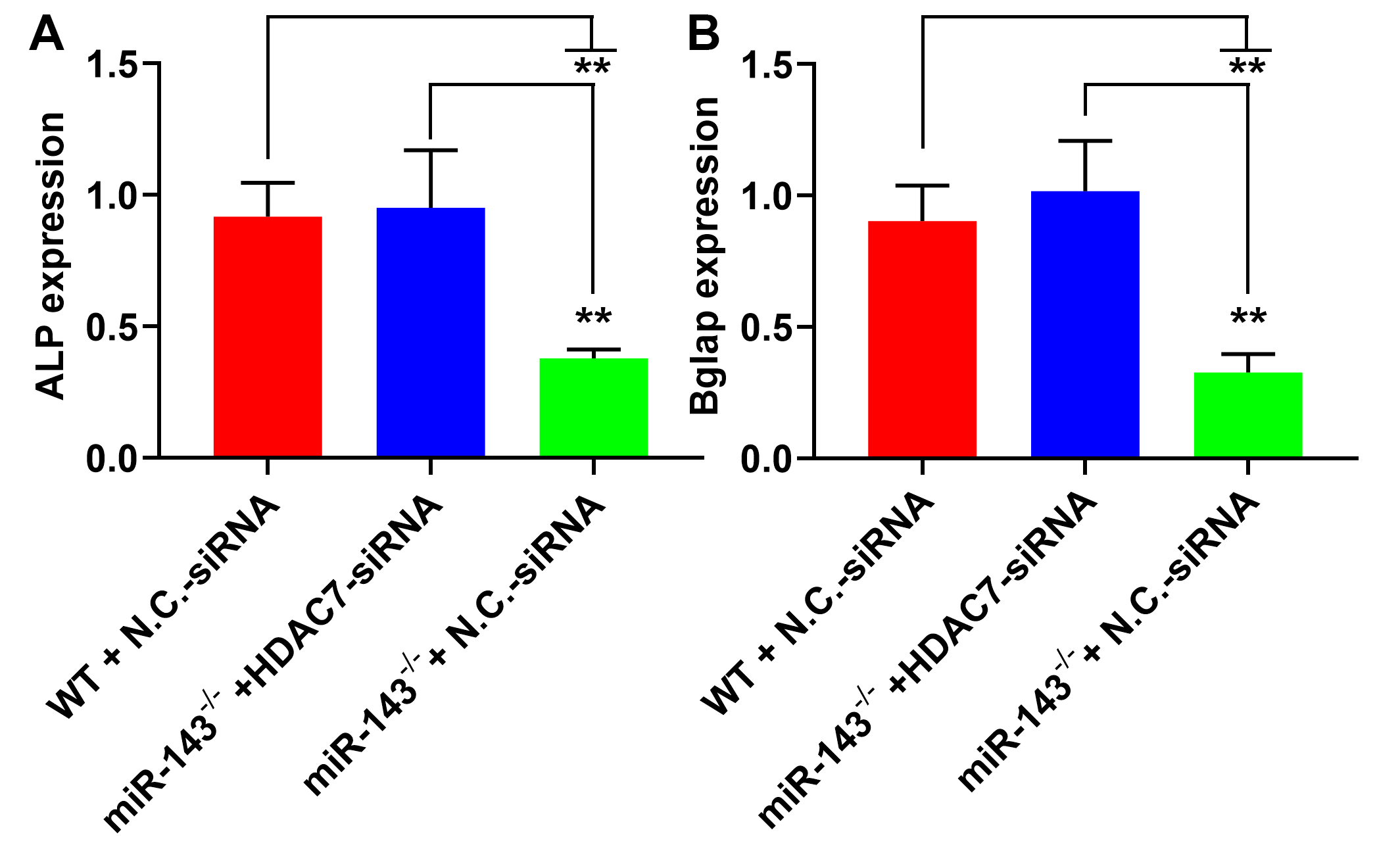

Supplement: Supplementary file 6 — Figure S5 [file 41419_2020_2377_MOESM6_ESM.tif]

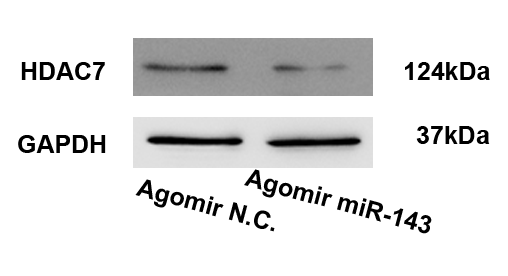

Supplement: Supplementary file 7 — Figure S6 [file 41419_2020_2377_MOESM7_ESM.tif]
